# Prediction of post-translational modification sites using multiple kernel support vector machine

**DOI:** 10.7717/peerj.3261

**Published:** 2017-04-27

**Authors:** BingHua Wang, Minghui Wang, Ao Li

**Affiliations:** 1 University of Science and Technology of China, School of Information Science and Technology, Hefei, China; 2 University of Science and Technology of China, Centers for Biomedical Engineering, Hefei, China

**Keywords:** Post-translational modification, Multiple kernels, Gaussian interaction profile kernel

## Abstract

Protein post-translational modification (PTM) is an important mechanism that is involved in the regulation of protein function. Considering the high-cost and labor-intensive of experimental identification, many computational prediction methods are currently available for the prediction of PTM sites by using protein local sequence information in the context of conserved motif. Here we proposed a novel computational method by using the combination of multiple kernel support vector machines (SVM) for predicting PTM sites including phosphorylation, O-linked glycosylation, acetylation, sulfation and nitration. To largely make use of local sequence information and site-modification relationships, we developed a local sequence kernel and Gaussian interaction profile kernel, respectively. Multiple kernels were further combined to train SVM for efficiently leveraging kernel information to boost predictive performance. We compared the proposed method with existing PTM prediction methods. The experimental results revealed that the proposed method performed comparable or better performance than the existing prediction methods, suggesting the feasibility of the developed kernels and the usefulness of the proposed method in PTM sites prediction.

## Introduction

Post-translational modifications (PTMs) refer to the covalent addition and enzymatic modifications of protein during or after protein biosynthesis, which play important roles in modifying protein functions and regulating gene expression (Mann & Jensen, 2003; Minguez et al., 2013; Walsh, 2006). Currently, a large amount of experimentally validated examples of PTMs have been detected. Among the general PTMs, protein phosphorylation principally on threonine (T), serine (S) or tyrosine (Y) sites is the primary PTM with a well-known role in a broad range of essential cellular processes such as translation, transcription, signal transduction and DNA repair (Li, Shakhnovich & Mirny, 2003; Matthews, 1995; Ubersax & Ferrell, 2007). In addition to phosphorylation, there are extensive studies describing experimental validated modifications on S/T/Y sites, such as acetylation, O-linked glycosylation (O-GalNAc, O-GlcNAc), sulfation and nitration (Blom et al., 2004; Hortin et al., 1986; Ischiropoulos, 2003; Mukherjee, Hao & Orth, 2007). Recent studies have explored that aforementioned types of PTM are involved in the majority of cellular activities and are related to various diseases (Huang et al., 2015; Li et al., 2010). In this respect, identification of potential PTM sites is important to understand the underlying molecular mechanisms for basic research and drug development.

During the past few decades, many efforts including experimental strategies and computational approaches have been undertaken to identify potential PTM sites (Fan et al., 2014; Gao et al., 2016; Xu et al., 2014a), and most of these methods used local sequence information for prediction due to the fact that PTMs generally occur at specific yet conserved motif in the target protein (Blom, Gammeltoft & Brunak, 1999; Eisenhaber & Eisenhaber, 2010; Miller & Blom, 2009). For example, to predict phosphorylation sites, a number of local sequence based tools have been developed, such as GPS 2.0 (Xue et al., 2008), Musite (Gao et al., 2010), PhosphoSVM (Dou, Yao & Zhang, 2014), NetPhos (Blom et al., 2004) and KinasePhos 2.0 (Wong et al., 2007). Besides phosphorylation, much effort also has been contributed to developing bioinformatics tools to identify other PTM sites. Gupta & Brunak, (2001) developed a prediction tool termed YinOYang which was trained using the local sequences of 40 O-GlcNAcylation sites. Later, a SVM-based model named O-GlcNAcPRED was developed for capturing potential O-GlcNAcylation sites (Jia, Liu & Wang, 2013). Meanwhile, Liu et al. (2011) provided the online service and local package of GPS-YNO2 1.0 for identification of tyrosine nitration with the previously developed GPS algorithm (Xue et al., 2008) and sequence information. Recently, Pan et al. (2014) developed a predictor termed GPS-TSP for the prediction of tyrosine sulfation with similar computational framework.

In addition to aforementioned methods, recently there is an increasing interest in predicting PTM sites that have potential functional relationships. For example, Qiu et al. (2016) proposed to predict different types of PTM on multiplex lysine (K) sites, which may have exceptional functions for basic research and drug development. Also, in consideration of the co-regulatory mechanism of lipid modifications, Xie et al. (2016) introduced a prediction tool that can investigate the co-regulatory in lipidation. Furthermore, in our previous study a computational approach was proposed for simultaneously predicting different types of PTM sites, by considering the context of *in situ* PTM that contained potential functional associations between multiple PTMs (Wang, Jiang & Xu, 2015). To this end, a network between target sites and corresponding modifications was constructed and further used as input features for prediction. The results suggested that existing relationships between target sites and different types of PTM was very helpful in predicting new PTM sites (Wang, Jiang & Xu, 2015).

Inspired by the aforementioned approaches, here we proposed a novel computational method by using the combination of multiple kernel support vector machines (SVM) for predicting PTM sites including phosphorylation, O-linked glycosylation, acetylation, sulfation and nitration. We developed a local sequence kernel and Gaussian interaction profile kernel to efficiently utilize local sequence information and site-modification relationships, respectively. Multiple kernels were further combined to train SVM for efficiently leveraging kernel information to boost predictive performance. The comparative analysis was based on ten-fold cross-validation process using collected datasets from several comprehensive sources. We compared the proposed method with existing PTM prediction methods such as PPSP (Xue et al., 2006), GPS 3.0 (Xue et al., 2008), Wang, Jiang & Xu (2015) and NetPhos (Blom et al., 2004) etc. The experimental results revealed that the proposed method achieved comparable or better performance than these state-of-the-art PTM sites prediction methods in terms of area under ROC (AUC) curve and other common measurements, demonstrating the feasibility of the developed kernels and the usefulness of the proposed method in PTM prediction.

## Method

### Data collection and preparation

We adopted a dataset of experimentally identified PTMs used in our previous study (Wang, Jiang & Xu, 2015), which included 2,990 S sites and 1,961 T sites (phosphorylation, O-linked glycosylation, acetylation) collected from several major comprehensive PTM databases, including dbPTM (version 3.0) (Lee et al., 2006), PhosphoSitePlus (Hornbeck et al., 2012), Phospho. ELM (Diella et al., 2004), dbOGAP (Wang et al., 2011) and SysPTM (Li et al., 2009). The detailed information of this dataset was provided in Wang, Jiang & Xu (2015). For Y sites, we followed the procedure described in Wang, Jiang & Xu (2015) and collected 1,791 local sequences (containing phosphorylation, sulfation and nitration) from dbPTM database (Lee et al., 2006) and the supplementary material provided by Pan et al. (2014). In these two datasets, the negative data contained the target sites that are not experimentally to be modified for a specific PTM or kinase group. To further confirm the fairness of constructing the negative dataset, we further randomly selected local sequences on S/T/Y sites from the known PTM proteins as additional negative samples, according to the median values of the positive samples of all PTMs or kinase groups, respectively. It should be noted that these additional negative samples were not experimentally to be modified by any PTMs or kinase groups. Finally, we totally obtained 3,239 local sequences on S sites, 2,037 local sequences on T sites and 1,880 local sequences on Y sites for this study. The detailed positive/negative information about each PTM or kinase group was illustrated in Table S1.

### Local sequence kernel similarity for target sites

After obtaining protein local sequences (10 upstream and 10 downstream residues and central residue has PTM) on S/T/Y sites, we computed the local sequence similarity for target sites using amino acid substitution matrix BLOSUM62, which has been proven to be efficient for calculating pairwise similarity (Gao et al., 2010). Then, the local sequence similarity between two samples *t*_*i*_ and *t*_*j*_ could be calculated as follows:

(1) \begin{eqnarray*}{S}_{seq} \left( {t}_{i},{t}_{j} \right) =\sum _{1\leq x\leq 21}BLOSUM62 \left( {t}_{i} \left( x \right) ,{t}_{j} \left( x \right) \right) \end{eqnarray*}

where *x* is the window size of a local sequence and is set to 21 in this study. ${t}_{i} \left( x \right) $ (or ${t}_{j} \left( x \right) $) represents the amino acid located in the *xth* position of *t*_*i*_ (or *t*_*j*_). Since the similarity between samples should be non-negative, we normalized *S*_*seq*_ using:

(2) \begin{eqnarray*}{K}_{seq} \left( {t}_{i},{t}_{j} \right) = \frac{{S}_{seq} \left( {t}_{i},{t}_{j} \right) -\min \nolimits \left( {S}_{seq} \right) }{\mathrm{max} \left( {S}_{seq} \right) -\min \nolimits \left( {S}_{seq} \right) } \end{eqnarray*}

where $\max \left( {S}_{seq} \right) /\min \left( {S}_{seq} \right) $ represents the largest/smallest number in the matrix, respectively. Thus, after applying this operation to the local sequence similarity, the matrix *K*_*seq*_ was obtained and could be considered as the local sequence kernel similarity, which was both symmetric and positive denite.

### Gaussian interaction profile kernel similarity for target sites

**Figure 1:**
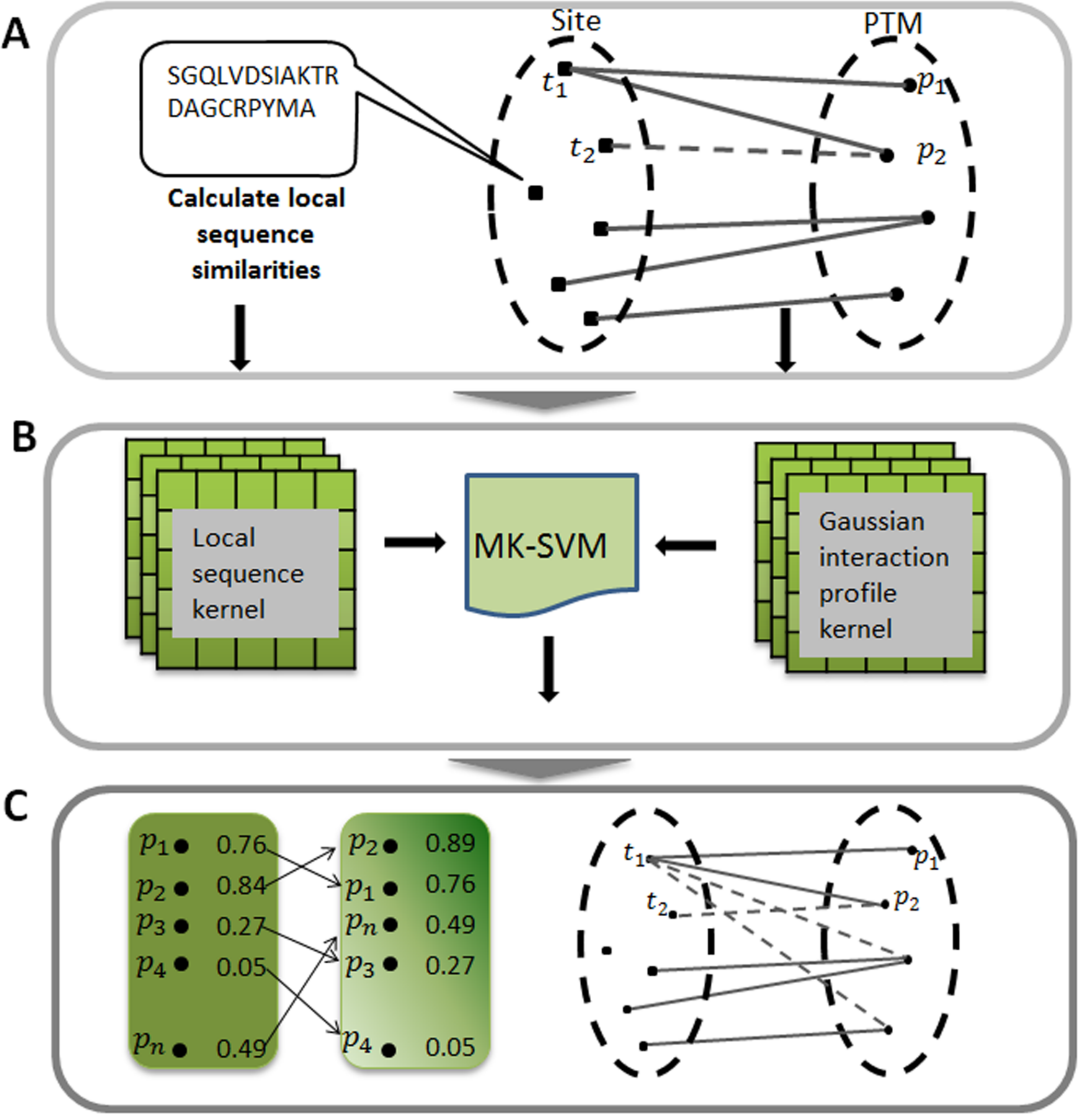
Illustration of predicting PTM sites. (A) Constructing bipartite network between target site and modification. (B) Calculating two kernels, namely the local sequence kernel and the Gaussian interaction profile kernel, and combining these two kernels to train SVM. (C) Ranking all the potential relationships between target site and modification. MK-SVM: multiple kernel SVM.

As described in Fig. 1A, the actual relationships between target sites and PTMs could be represented as a bipartite network. Formally, given a set $T= \left\{ {t}_{1},{t}_{2},\ldots ,{t}_{m} \right\} $ of sites and $P= \left\{ {p}_{1},{p}_{2},\ldots ,{p}_{n} \right\} $ of PTMs, and an edge $E= \left\{ {e}_{ij},{t}_{i}\in T,{p}_{j}\in P \right\} $ was drawn in the network if the target site *t*_*i*_ has been experimentally modified by this PTM *p*_*j*_. For simplicity, we can further characteristic this bipartite network by an adjacency matrix *A*, in which each row denotes target sites and each column denotes different types of PTM. The entity $A \left\{ i,j \right\} $ in row *i* and column *j* equals 1 if the target site *t*_*i*_ is modified by the PTM *p*_*j*_, otherwise 0. Here, *m* is the number of target sites, and *n* is the category of the PTM types.

Due to the fact that the row *i* of adjacency matrix *A* indicates the interaction profile of a target site *t*_*i*_ (or *t*_*j*_), which specifies the presence or absence of relationship with every PTM in the constructed bipartite network, we adopted a powerful kernel named Gaussian interaction profile kernel (GIP) that has been widely used in the area of drug-target interaction prediction (Van Laarhoven, Nabuurs & Marchiori, 2011). The definition of Gaussian kernel between target sites was using the follow equation:

(3) \begin{eqnarray*}{K}_{GIP} \left( {t}_{i},{t}_{j} \right) =\exp \nolimits \left( -\gamma { \left\| {A}_{ti}-{A}_{tj} \right\| }^{2} \right) \end{eqnarray*}

where *A*_*ti*_ (or *A*_*tj*_) represents the interaction profile for the target site *t*_*i*_ (or *t*_*j*_), namely the binary vector encoding relationship between sites *t*_*i*_ (or *t*_*j*_) and each PTM. $ \left\| \cdot \right\| $ indicates the Euclidean distance between *A*_*ti*_ and *A*_*tj*_ andthe parameter *γ* is the kernel bandwidth. Generally, the kernel bandwidth can be obtained by cross-validation process, here in this study was set to 0.001. Finally, the Gaussian interaction profile kernel similarity for target sites, denoted by *K*_*GIP*_, is an m by m symmetric matrix. It should be noted that *K*_*GIP*_ should be recalculated since the adjacency matrix *A* changed when performing cross-validation process.

### Multiple kernel SVM

In this section first we briefly introduced the concepts of SVM for classification tasks. The detailed information were also provided in Huang & Wang (2006) and Vapnik (2000). Given a training dataset $T= \left\{ \left( {x}_{1},{y}_{1} \right) , \left( {x}_{2},{y}_{2} \right) ,\ldots , \left( {x}_{n},{y}_{n} \right) \right\} ,{x}_{i}\in {R}^{m}$ and ${y}_{i}\in \left\{ +1,-1 \right\} $. For SVM with L1 soft margin formulation, we can use following equation to deal with the primal problem:

(4) \begin{eqnarray*}\text{}\min \nolimits J \left( \vec{\mathrm{w}},\vec{\xi } \right) = \frac{1}{2} \parallel \vec{\mathrm{w}}{\parallel }^{2}+C\sum _{i=1}^{n}{\xi }_{i}& \end{eqnarray*}

(5) \begin{eqnarray*}\mathrm{s}.\mathrm{t}. {y}_{i} \left( {\vec{\mathrm{w}}}^{T}\phi \left( \vec{{x}_{i}} \right) +b \right) \geq 1-{\xi }_{i},\quad i=1\ldots n& \end{eqnarray*}

\begin{eqnarray*}\quad {\xi }_{i}\geq 0& \end{eqnarray*}

where *ξ*_i_ ≥ 0 represent the non-negative slack variables and *C* is the regularization parameter. The aforementioned quadratic optimization problem could be solved by using the Lagrange function and differentiating with respect to $\vec{\mathrm{w}},b$ and *ξ*_i_, then the primal problem would transform to the dual problem:

(6) \begin{eqnarray*}\text{}& \max \nolimits \sum _{j=1}^{n}{a}_{i}- \frac{1}{2} \sum _{i,j=1}^{n}{a}_{i}{a}_{j}{y}_{i}{y}_{j}K \left( \vec{{x}_{i}},\vec{{x}_{j}} \right) \end{eqnarray*}

(7) \begin{eqnarray*}\mathrm{s}.\mathrm{t}. \sum _{i=1}^{n}{y}_{i}{a}_{i}& =0\end{eqnarray*}

\begin{eqnarray*}\quad 0\leq {a}_{i}\leq C, i& =1\ldots n. \end{eqnarray*}

In a classification task, the optimal ${\vec{a}}^{\mathrm{\ast }},{\vec{w}}^{\mathrm{\ast }},{b}^{\mathrm{\ast }}$ would be obtained and the final predictive model can be represented as:

(8) \begin{eqnarray*}{y}_{i} \left( \sum _{j=1}^{n}{a}_{j}^{\ast }{y}_{j}K \left( \vec{{x}_{i}},\vec{{x}_{j}} \right) +{b}^{\ast } \right) =1,\quad i=1\ldots n\end{eqnarray*}

where $K \left( \vec{{x}_{i}},\vec{{x}_{j}} \right) =\phi \left( \vec{{x}_{i}} \right) \phi \left( \vec{{x}_{j}} \right) $ is the kernel function.

Here multiple kernels namely local sequence kernel and Gaussian kernel were integrated into the kernel function and was described as follows:

(9) \begin{eqnarray*}K \left( \vec{{x}_{i}},\vec{{x}_{j}} \right) =\sum _{d=1}^{m}{\beta }_{d}{K}_{d} \left( \vec{{x}_{i}},\vec{{x}_{j}} \right) ,\quad {\beta }_{d}\geq 0\end{eqnarray*}

where *m* = 2, *K*_1_ and *K*_2_ were local sequence kernel and Gaussian kernel, respectively. In this study, we defined the integrated kernel *K* as the custom kernel function, instead of using the default kernel of SVM. We used LIBSVM (v.3.17) (Chang & Lin, 2011) SVM implementation freely available for the MATLAB environment. In applying the SVM algorithm to our dataset, we used balanced penalization in the case of positive and negative training dataset of different sizes. In all experiments, we used the default *C* regularization parameter. The whole procedure of this work was illustrated in Fig. 1.

### Performance assessment

In this study, ten-fold cross-validation as described in existing studies (Gao et al., 2010; Xu et al., 2014a; Xue et al., 2006) was applied to assess the predictive performance of the proposed method. For a given PTM, 9/10 randomly chosen samples were used as the training data while the remaining 1/10 were used as the test data. The ten-fold cross-validation tests were repeated 10 times. As a result, the original data set was covered successfully both in the training and in the test data. The final evaluation was based on the average of these ten performances. Receiver-operating characteristic (ROC) curve, which plots true positive rate (sensitivity, *Sn*) against false positive rate (1-specificity, 1 − *Sp*) by gradually changing different thresholds, was utilized to estimate the predictive ability of the method. *Sn* is defined as the proportion of true positives that are correctly observed by the classifier, whereas *Sp* is given by the proportion of true negatives that are correctly identified. The corresponding area under ROC curve namely AUC is also calculated, with AUC = 1 represents perfect performance and 0.5 means random performance. In addition, other conventional measurements such as precision (Pre), accuracy (Acc) and Matthews’s correlation coefficient (MCC) were also applied to assess the predictive performance, and the definitions were shown as below:

(10) \begin{eqnarray*}\text{}Sn= \frac{TP}{TP+FN} & \end{eqnarray*}

(11) \begin{eqnarray*}Sp= \frac{TN}{TN+FP} & \end{eqnarray*}

(12) \begin{eqnarray*}Pre= \frac{TP}{TP+FP} & \end{eqnarray*}

(13) \begin{eqnarray*}Acc= \frac{TP+TN}{TP+TN+FP+FN} & \end{eqnarray*}

(14) \begin{eqnarray*}MCC= \frac{TP\times TN-FP\times FN}{\sqrt{ \left( TP+FN \right) \times \left( TP+FP \right) \times \left( TN+FN \right) \times \left( TN+FP \right) }} & \end{eqnarray*}

where *TP*, *TN*, *FP* and *FN* refer to true positives, true negatives, false positives and false negatives, respectively.

## Results

### Comparison with existing methods for phosphorylation

**Figure 2:**
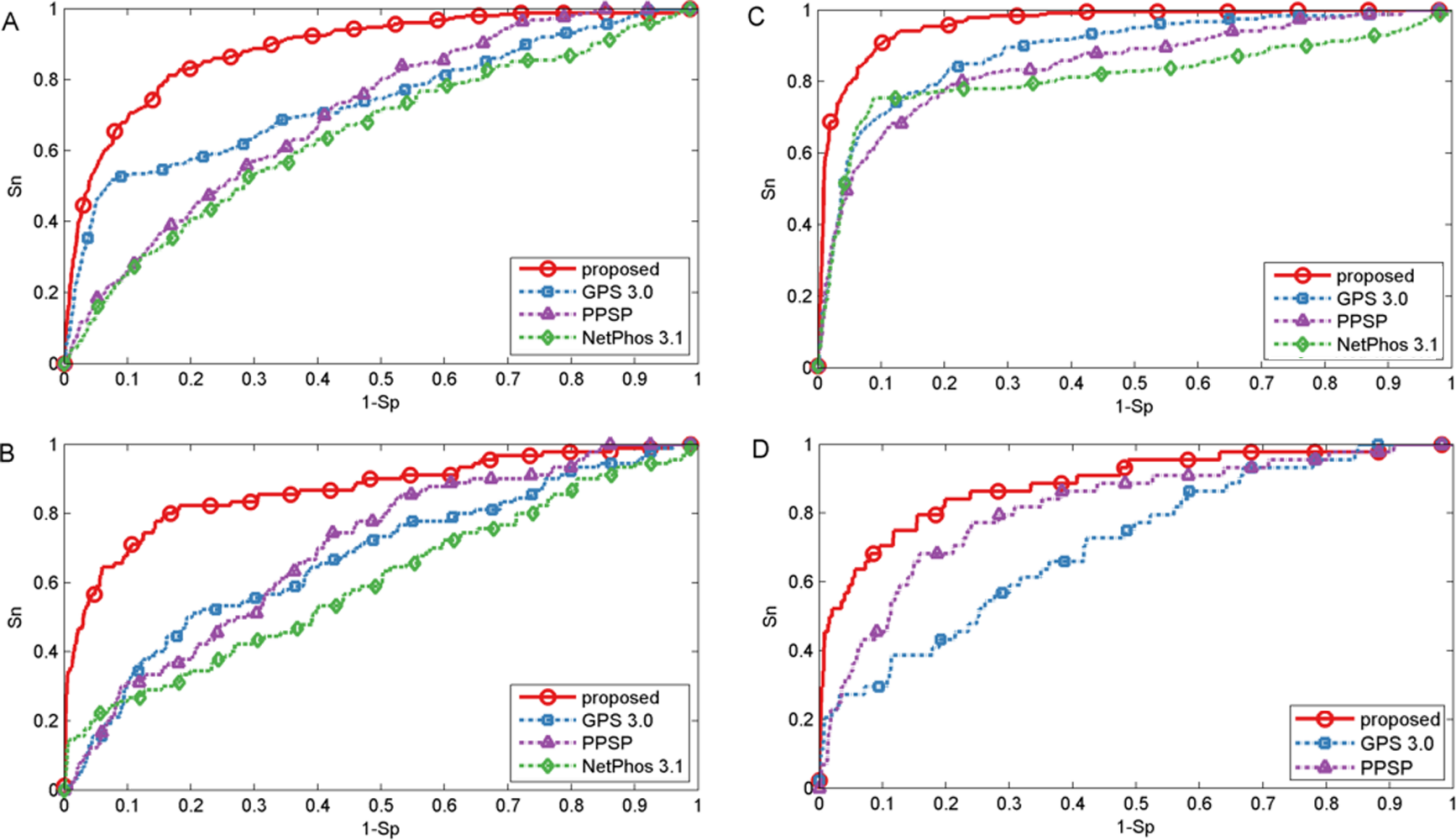
Performance of phosphorylation ROC curves for kinase groups CAMK, CK1, CMGC, and TKL with different methods. The kinase groups CAMK (A) and CK1 (B) are in response to S sites, and the kinase groups CMGC (C) and TKL (D) are in response to T sites. The red, blue, purple and green lines represent the performance of the proposed method, GPS, PPSP, NetPhos, respectively.

To evaluate the power of the proposed method, first three common phosphorylation prediction methods, PPSP (Xue et al., 2006), GPS (version 3.0) (Xue et al., 2008) and NetPhos (version 3.1) (Blom et al., 2004) were used to make comparison. We took kinase groups CAMK, CMGC, CK1 and TKL as examples to illustrate the predictive performance. It should be stated that the ten-fold cross-validation process is not available for GPS and NetPhos, so the phosphorylation dataset was utilized as testing dataset to evaluate the predictive performance, which may lead to over-estimation of the predictive performance of these tools. However, our proposed method still obtained promising and competitive performance. The ROC curves were plotted for four methods to compare the predictive performance at each specificity level and displayed in Fig. 2. As shown in Fig. 2, the proposed method achieved significantly better overall performance for four kinase groups than all other prediction methods. Performance of other kinase groups on *S*∕*T* and Y sites were displayed in Figs. S1 and S2, respectively.

Besides the ROC curves, the corresponding AUC value for each phosphorylation kinase group on S/T/Y sites was also calculated for each method and displayed in Table 1. It was indicated that our proposed method was consistently better than GPS, PPSP and NetPhos. For example, the AUC achieved by the proposed method for kinase group CAMK on S sites was 14.7%, 24.2% and 18.2% higher than GPS, PPSP and NetPhos, respectively. For kinase group TKL on T sites, the corresponding AUC values were 88.6%, 71.2% and 81.4% for the proposed method, GPS and PPSP, respectively. Also, from Table 1 it can be seen that our proposed method achieved comparable or better performance than Wang, Jiang & Xu (2015) that also used both sequence information and site-modification relationships, demonstrating the feasibility and usefulness of the developed kernels in predicting PTM sites. In addition, in order to ensure that the redundancy between training and evaluation data was minimized, all protein sequences were grouped into ten sets using BLASTClust by following Dou, Yao & Zhang (2014). Then, the proposed method was compared with PPSP and Wang, Jiang & Xu (2015) by cross validation of these grouped ten sets. For GPS and NetPhos, cross validation process is not supported as they only provide the web servers to make prediction. The results listed in Table S2 indicated that our proposed method was also consistently better than PPSP and in general comparable to Wang, Jiang & Xu (2015). Taken together, the proposed method achieved comparable or better performance for the prediction of phosphorylation sites.

**Table 1:** Comparison of AUC values with different methods for phosphorylation kinase groups on S, T and Y sites.

Sites	Kinase group	Proposed (%)	GPS 3.0 (%)	PPSP (%)	NetPhos 3.1 (%)	Wang, Jiang & Xu (2015) (%)
S	AGC	90.0	72.1	80.3	66.2	89.8
	CAMK	88.8	74.1	64.6	70.6	87.7
	CK1	86.7	67.1	69.5	59.9	87.9
	CMGC	91.8	82.1	81.5	65.6	91.7
	STE	92.2	64.4	70.6	–	91.3
	TKL	89.4	99.6	69.0	–	91.9
	Atypical	92.7	–	72.8	64.2	92.6
	Other	89.3	–	78.2	–	87.1
T	AGC	92.7	77.0	74.6	68.3	92.5
	CAMK	89.5	82.2	74.3	64.0	87.2
	CK1	92.2	57.1	80.9	53.7	92.3
	CMGC	96.2	88.5	84.7	81.5	95.5
	STE	94.7	73.7	79.0	–	93.4
	TKL	88.6	71.2	81.4	–	85.1
	Atypical	91.6	–	67.1	62.4	89.5
	Other	83.3	–	70.2	–	80.8
Y	TK	97.1	90.9	78.5	69.1	96.8
	CMGC	98.1	87.2	86.9	–	96.9
	STE	95.4	86.2	79.4	–	94.4
	TKL	89.1	81.2	76.8	–	87.7
	Other	76.1	–	64.0	–	73.4

**Figure 3:**
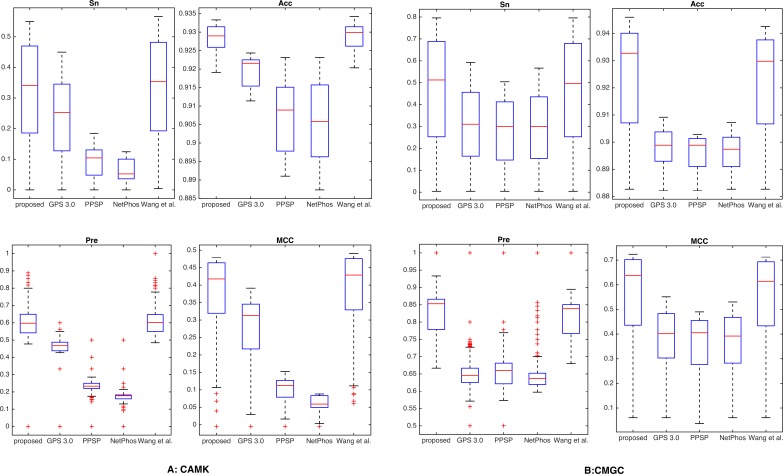
Comparison with different methods of *Sn*, Acc, Pre and MCC on the phosphorylation kinase groups CAMK and CMGC. The Sn, Acc, Pre and MCC value comparison with different methods for kinase groups CAMK (A) and CMGC (B) at the medium stringency level (*Sp* = 95.0%, with corresponding threshold of 5.9e−4 and 2.0e−3, respectively). The horizontal axis represents the proposed method, GPS, PPSP, NetPhos and Wang, Jiang & Xu (2015) respectively.

Additionally, by following the study of Fan et al. (2014), a threshold was set for each method such that the specicity of each method was equal to 95.0% (medium) or 99.0% (high). We took two kinase groups (CAMK and CMGC) as examples, and the corresponding measurements were computed and reported in Fig. 3. It suggested that the proposed method achieved comparable or better predictive performance than other prediction methods in all cases. For instance, with *Sp* = 95.0%, *Sn*, Acc, Pre and MCC values of kinase group CMGC on T site were increased by 29.1%, 3.51%, 10.3% and 21.7% compared with PPSP and had an improvement of 20.4%, 2.41%, 6.76% and 14.7% compared with GPS respectively. In addition, for kinase groups CAMK and CMGC, the precision values obtained by our proposed method were 59.8% and 85.1%, and the precision values of Wang, Jiang & Xu (2015) were 59.7% and 84.2%, respectively. Table S3 showed the detailed comparative results for kinase groups CAMK and CMGC with *Sp* = 99.0%. From this Table, we can see that our proposed method obtained better performance than other prediction methods, especially sensitivity. For example, for kinase group CMGC the proposed method obtained the *Sn* value of 50.4%, while the *Sn* values of GPS, PPSP, NetPhos and Wang, Jiang & Xu (2015) were 16.3%, 13.7%, 15.0% and 38.7%, respectively. Besides, PTMPred (Xu et al., 2014b) was also used to make comparison and the results were illustrated in Table S4, indicating that the proposed method compared favorably with it.

**Figure 4:**
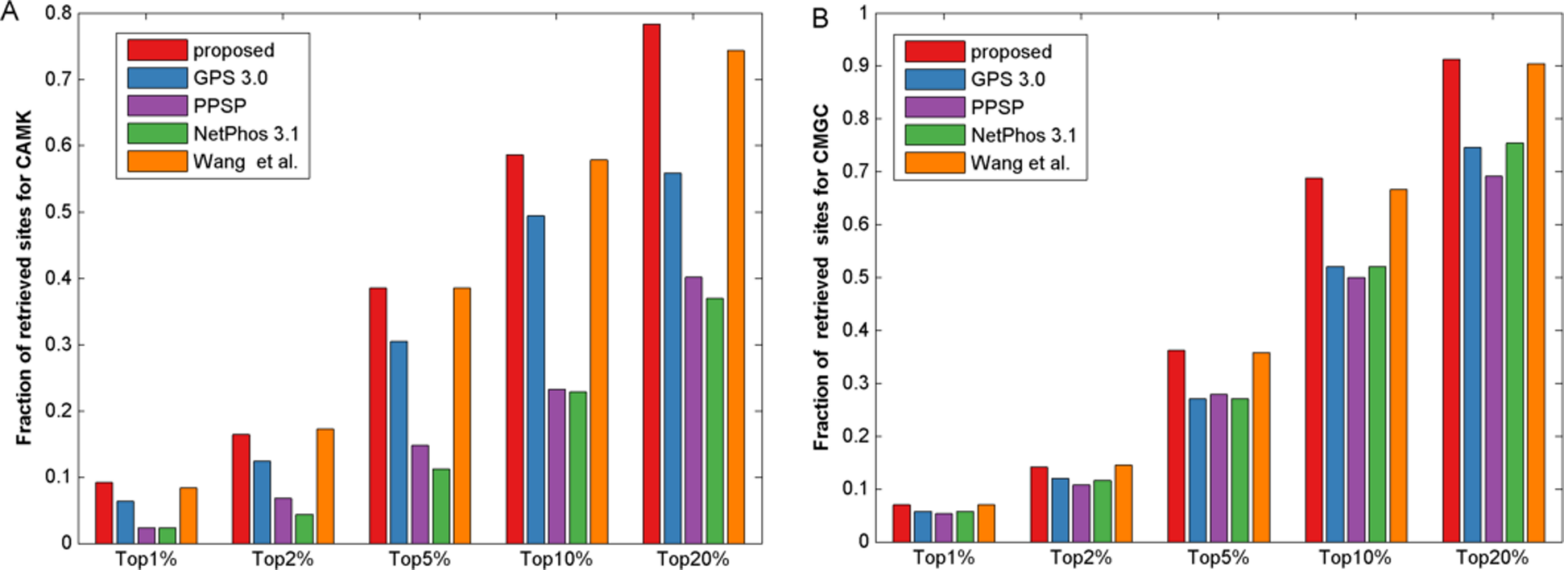
The fraction of retrieved sites for kinase groups CAMK and CMGC. (A) represents the performance of CAMK, and (B) represents the performance of CMGC. The horizontal axis represents five top 1, 2, 5, 10 and 20% of the total samples.

It is known that the control of false positive prediction results is usually critical in the field of computational bioinformatics (Xu & Wang, 2015). Hence, in addition to the aforementioned measurements, we used similar bar plot with those adopted in previous studies (Peng & Li, 2016; Xu & Wang, 2015) to indicate the number of true positives in top-ranked results. For each percentile *p*%, first we counted the number of true positives in the top ranked *p*%*total samples, then we calculated the fraction of true positives by dividing total positive samples. Here we took CAMK and CMGC for instance, results of five top 1, 2, 5, 10 and 20 percent of the total samples were compared (Fig. 4). It was observed that the proposed method gave most of the known sites higher ranks than other prediction methods investigated in this study. For example, for kinase group CAMK at the top20%, the fraction of true positives of the proposed method was 78.3% and the corresponding values of GPS, PPSP, NetPhos and Wang, Jiang & Xu (2015) were 55.8%, 40.2%, 36.9% and 74.3%, respectively. Also, Fig. S3 suggested that our method had comparable fraction of predicted sites with other prediction methods. In summary, the proposed method can obtain comparable or better performance for the prediction of phosphorylation sites.

### Comparison with existing methods for other PTMs

**Figure 5:**
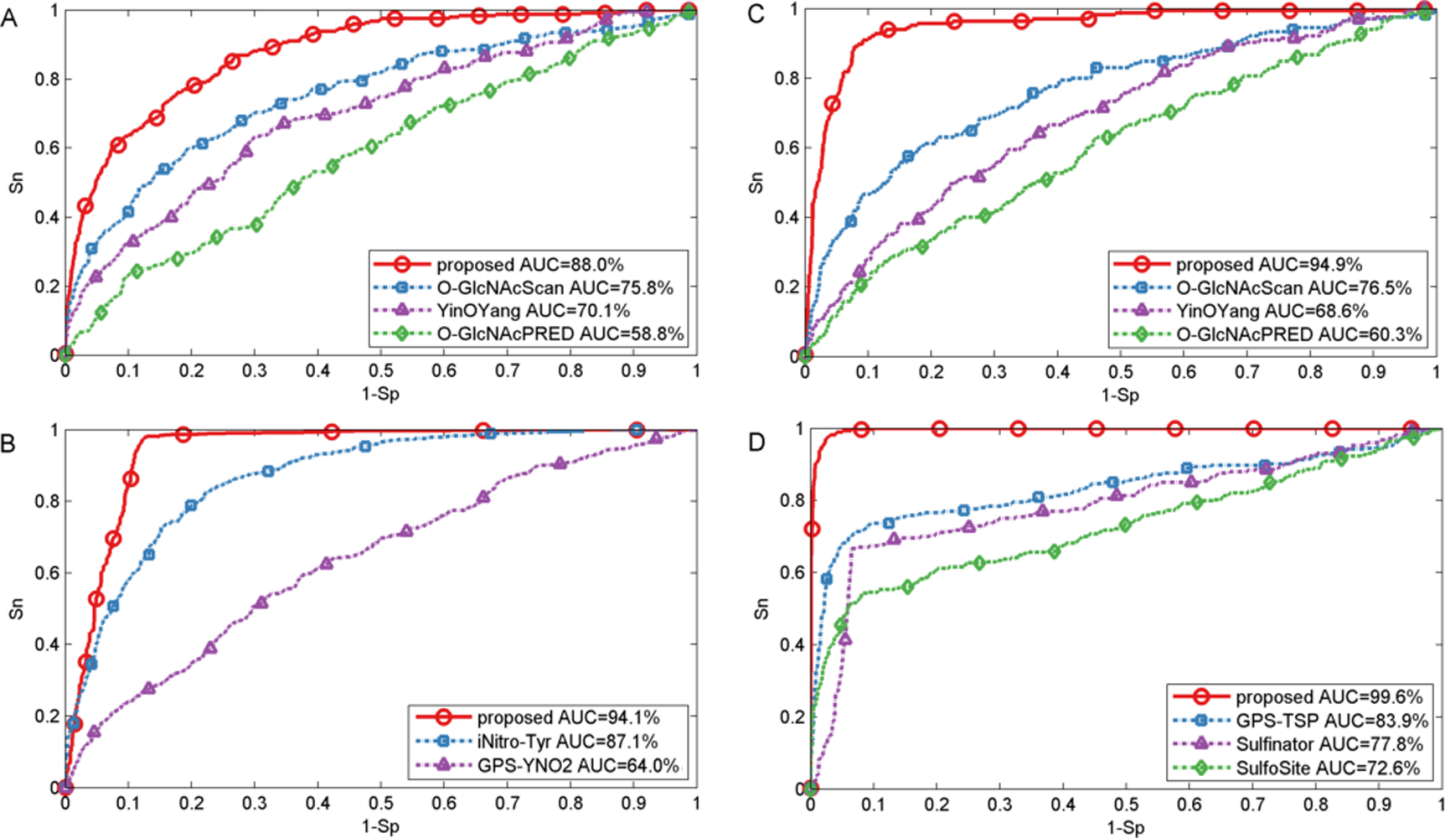
Performance of ROC curves for O-GlcNAc, nitration and sulfation with different methods. (A) The performance of O-GlcNAc on S sites, (B) the performance of nitration on Y sites, (C) the performance of O-GlcNAc on T sites, and (D) the performance of sulfation on Y sites.

In this section, we also made comparison with existing methods about other PTMs. For O-GlcNAc the proposed method was compared with several methods including YinOYang (Gupta & Brunak, 2001), O-GlcNAcPRED (Jia, Liu & Wang, 2013) and O-GlcNAcScan (Wang et al., 2011). The detailed ROC curves of different methods were illustrated in Fig. 5. The proposed method achieved an AUC value of 88.0%, and the corresponding AUC values of YinOYang, O-GlcNAcPRED and O-GlcNAcScan were 70.1%, 58.8% and 75.8% on S sites respectively (Fig. 5A). In addition, the AUC values achieved by the proposed method also had an improvement by 26.3%, 34.6% and 18.4% compared with YinOYang, O-GlcNAcPRED and O-GlcNAcScan, respectively on T sites (Fig. 5C). Therefore, the proposed method remarkably outperformed the predictive performance compared with YinOYang, O-GlcNAcPRED and O-GlcNAcScan on both S and T sites. Besides, we also studied the predictive performance of nitration and sulfation on Y sites compared with other existing methods. For sulfation, GPS-TSP (Pan et al., 2014), Sulfinator (Monigatti et al., 2002) and SulfoSite (Chang et al., 2009) were applied to compare the predictive performance, while GPS-YNO2 (Liu et al., 2011) and iNitro-Tyr (Xu et al., 2014c) were compared with the proposed method for nitration. As shown in Fig. 5D, for sulfation, the AUC values were increased by 15.7% compared with GPS-TSP. For nitration (Fig. 5B) the AUC value of proposed method was 7.0% and 30.1% higher than iNitro-Tyr and GPS-YNO2, respectively. Furthermore, the comparison of *Sn*, Acc, Pre and *Sp* with multiple types of PTM at the two stringency levels was listed in Table 2. Taking O-GlcNAc on S sites as an example, our proposed method obtained a precision of 45.1% at *Sp* = 95.0%, while the precision values of O-GlcNAcScan, O-GlcNAcPRED and YinOYang were 34.5%, 26.8% and 14.3%, respectively. For O-GlcNAc on T sites, the *Sn* value of our proposed method was 28.5% at *Sp* = 99.0%, while the corresponding values of O-GlcNAcScan, O-GlcNAcPRED and YinOYang were 10.9%, 4.85% and 3.03%, respectively. For sulfation on Y sites, with *Sp* = 95.0%, the precision value of the proposed method was 77.1% and the corresponding precision values of GPS-TSP, Sulfinator and SulfoSite were 69.4%, 54.3% and 60.9%, respectively. For nitration on Y sites, with *Sp* = 99.0%, the precision value was increased by 14.3% compared with GPS-YNO2, while was 1.7% lower than iNitro-Tyr, respectively. However with *Sp* = 95.0%, all measurements were higher than other prediction methods. In conclusion, aforementioned analyses suggested that proposed method outperformed other prediction methods in predicting multiple types of PTM on serine, threonine and tyrosine sites.

**Table 2:** For other PTMs, performance comparison of different methods on S/T/Y sites at the high (*Sp* = 99.0%) and median stringency level (*Sp* = 95.0%).

PTMs	Methods	*Sp* (%)	*Sn* (%)	Pre (%)	Acc (%)	*Sp* (%)	*Sn* (%)	Pre (%)	Acc (%)
S: O-GlcNAc	Proposed	99.0 (threshold: 7.6e−4)	24.3	66.3	93.4	95.0 (threshold: 6.1e−4)	50.6	45.1	91.7
	O-GlcNAcScan	99.0	16.1	56.5	92.7	95.0	32.5	34.5	90.3
	O-GlcNAcPRED	99.0	11.1	47.4	92.4	95.0	22.6	26.8	89.5
	YinOYang	99.0	3.29	21.1	91.8	95.0	10.3	14.3	88.6
T: O-GlcNAc	Proposed	99.0 (threshold: 4.4e−3)	28.5	71.2	93.3	95.0 (threshold: 1.8e−3)	75.1	56.9	93.4
	O-GlcNAcScan	99.0	10.9	48.6	91.8	95.0	33.0	37.3	90.0
	O-GlcNAcPRED	99.0	4.85	29.6	91.4	95.0	15.8	21.6	88.5
	YinOYang	99.0	3.03	20.8	91.2	95.0	11.5	7.74	88.2
Y: Nitration	Proposed	99.0 (threshold: 2.2e−2)	10.3	93.2	48.8	95.0 (threshold: 1.3e−2)	53.1	93.2	71.3
	iNitro-Tyr	99.0	15.8	94.9	51.9	95.0	40.2	91.2	64.0
	GPS-YNO2	99.0	2.82	78.9	44.6	95.0	16.2	80.7	50.4
Y: Sulfation	Proposed	99.0 (threshold: 1.2e−2)	90.8	93.9	93.9	95.0 (threshold: 3.8e−3)	98.9	77.1	95.9
	GPS-TSP	99.0	33.7	85.2	89.5	95.0	67.4	69.4	90.9
	Sulfinator	99.0	4.76	39.4	85.1	95.0	35.2	54.3	86.3
	SulfoSite	99.0	24.9	80.9	88.2	95.0	45.8	60.9	87.8

### Analysis of the predicted potential PTM sites

Due to the difficulty of the experimental verification, the computational method is required to have the ability to detect unknown PTM sites (Xu & Wang, 2015). Hence, we extracted the top ten ranked candidate sites which were not experimentally modified by acetylation or O-GalNAc in our dataset according to the probability estimates of LIBSVM package, respectively. We manually checked these predicted results from UniProtKB database (Boutet et al., 2007) and literature. Table 3 showed the detailed top ten predicted results of acetylation, in which we found that some sites of proteins have been demonstrated to be modified by acetylation. The potential acetylation site with largest probability (0.771) was Thr2 of EBP. Interestingly, we found that this site can be modified by acetylation in the UniProtKB database (http://www.uniprot.org/uniprot/Q15125#ptm_processing). At the same time, in Table S5, we also listed the top ten ranked candidate sites for O-GalNAc. Interestingly, we found according to previous study (Carlsson, Lycksell & Fukuda, 1993) that the Ser207 of protein LAMP2 (probability: 0.702) could be modified by O-GalNAc. These results further demonstrated the proposed method had the ability to discover new target sites, which could be helpful for the subsequent experimental verification.

**Table 3:** Information on top 10 potential candidate sites for acetylation.

Ranking	UniProt ID	Protein name	Position	Probability
1	Q15125	EBP	2	0.771
2	Q96KX2	CAPZA3	2	0.550
3	P46734	MAP2K3	222	0.342
4	Q15125	EBP	3	0.245
5	Q00987	MDM2	4	0.197
6	P68431	HIST1H3A	4	0.195
7	P45985	MAP2K4	261	0.193
8	O14733	MAP2K7	275	0.105
9	P21453	S1PR1	4	0.097
10	P53779	MAPK10	221	0.031

## Discussion and Conclusion

Protein post-translational modifications play an important role in multiple biological processes, and have an intimate relationship with many diseases. Thus, identification of potential PTM sites is important to promote our understanding of underlying PTM regulatory mechanisms. Considering the high-cost and labor-intensive of experimental identification, there is an urgent need to develop effective and fast computational methods for PTM sites identification. Hence, in this work, we introduced a computational approach by using the combination of multiple kernels based on support vector machines (SVM) for predicting PTM sites. To efficiently incorporate the local sequence information and existing site-modification relationships, we calculated two kernels; namely, the local sequence kernel and the Gaussian interaction profile kernel, respectively. Upon ten-fold cross validation process using the PTM dataset on S/T/Y sites, the proposed method had a better or comparable performance than other existing prediction methods, indicating that multiple kernels could be very useful for the prediction of PTM sites. Furthermore, through the analysis of the highly ranked results, we found some important predicted potential PTM sites which had been confirmed by UniProtKB database and literature. It is anticipated that these ranked results can be helpful for biological research and experimental validations by providing important clues of the PTM mechanism.

The improvement of the proposed method could be attributed to a combination of several factors. First, kernel based methods might derive high performance from the ability to incorporate biological information via a suitable kernel function, which transforms data points embedding them into a higher dimensional space (Conforti & Guido, 2010). Second, different kernels may be using inputs coming from different representations possibly from multiple information sources or modalities (Gönen & Alpaydın, 2011). Combining kernels is one possible way to combine multiple information sources (Gönen & Alpaydın, 2011). Thus, multiple kernels are combined to train SVM for efficiently leveraging different kernels information to boost predictive performance. Further, the combination of multiple kernels possibly increases the generalization of the model, which usually leads to better performance, since the model can benefit from different heterogeneous information sources in a systematic way (Nascimento, Prudêncio & Costa, 2016). Of course, our proposed method still has some limitations in identifying PTM sites. First, we only took into consideration protein local sequence information, while other important biological functional information such as gene-ontology (GO) and protein-protein interactions (PPI) can be further combined into the predictive method. Second, currently available site-modification relationships are still limited in databases, it is anticipated that the performance of the predictive method would be further enhanced when more site-modification relationships become available in the future.

##  Supplemental Information

10.7717/peerj.3261/supp-1 Supplemental Information 1 Supplementary material

10.7717/peerj.3261/supp-2 Supplemental Information 2 Code and data

## Additional Information and Declarations

### Data Availability

The following information was supplied regarding data availability: The raw data has been supplied as Supplementary Files.
