# Supplementary material for: Prediction of post-translational modification sites using multiple kernel support vector machine
Source: PeerJ. 2017 Apr 27;5:e3261. doi: 10.7717/peerj.3261 (PMC5410141; doi:10.7717/peerj.3261)

**Supplementary Material**

**Table S1**. The detailed positive/negative information about each PTM or kinase group on S/T/Y sites.

| **Kinase group/PTM** | **S** | | **T** | | **Y** | |
| --- | --- | --- | --- | --- | --- | --- |
|  | **positive** | **negative** | **positive** | **negative** | **positive** | **negative** |
| **TKL** | 15 | 3224 | 44 | 1993 | 83 | 1797 |
| **AGC** | 640 | 2599 | 163 | 1874 | - | - |
| **Atypical** | 212 | 3027 | 57 | 1980 | - | - |
| **CAMK** | 249 | 2990 | 76 | 1961 | - | - |
| **CK1** | 90 | 3149 | 43 | 1994 | - | - |
| **CMGC** | 642 | 2597 | 240 | 1797 | 53 | 1827 |
| **STE** | 163 | 3076 | 48 | 1989 | 66 | 1814 |
| **Other** | 322 | 2917 | 82 | 1955 | 89 | 1791 |
| **O-GalNAc** | 837 | 2402 | 1261 | 776 | - | - |
| **O-GlcNAc** | 243 | 2996 | 165 | 1872 | - | - |
| **Acetylation** | 472 | 2767 | 64 | 1973 | - | - |
| **TK** | - | - | - | - | 466 | 1414 |
| **nitration** | - | - | - | - | 1062 | 818 |
| **sulfation** | - | - | - | - | 273 | 1607 |

**Table S2**. Comparison of AUC values between the proposed method and other methods for kinase groups on S/T sites by cross validation of BLASTClust-grouped data sets.

| sites | Kinase groups | proposed(%) | PPSP(%) | Wang et al.(%) |
| --- | --- | --- | --- | --- |
| S | AGC | 90.0 | 79.4 | 86.2 |
|  | Atypical | 92.9 | 72.7 | 92.8 |
|  | CAMK | 86.7 | 69.1 | 86.2 |
|  | CK1 | 82.4 | 67.1 | 83.8 |
|  | CMGC | 91.7 | 81.0 | 89.3 |
|  | STE | 88.8 | 68.0 | 88.7 |
|  | Other | 83.6 | 73.9 | 84.8 |
|  | TKL | 64.1 | 25.9 | 71.2 |
| T | AGC | 88.9 | 72.8 | 89.2 |
|  | Atypical | 87.0 | 63.3 | 83.3 |
|  | CAMK | 86.7 | 73.4 | 84.8 |
|  | CK1 | 92.1 | 78.0 | 92.8 |
|  | CMGC | 93.7 | 82.6 | 94.5 |
|  | STE | 91.8 | 65.0 | 86.4 |
|  | Other | 76.7 | 63.5 | 78.7 |
|  | TKL | 88.1 | 73.2 | 85.6 |

**Table S3**. Performance comparison of different methods for kinase groups CAMK and CMGC at the high stringency level (Sp = 99.0%).

| Kinase groups | Methods | Sp(%) | Sn(%) | Acc(%) | Pre(%) | MCC |
| --- | --- | --- | --- | --- | --- | --- |
| CAMK | proposed | 99.0(threshold:8.5e-4) | 22.5 | 93.1 | 65.1 | 0.356 |
|  | GPS 3.0 | 99.0 | 10.4 | 92.2 | 46.4 | 0.193 |
|  | PPSP | 99.0 | 2.81 | 91.6 | 18.9 | 0.045 |
|  | NetPhos 3.1 | 99.0 | 2.81 | 91.7 | 20.6 | 0.049 |
|  | Wang et al. | 99.0 | 20.5 | 92.9 | 63.7 | 0.334 |
| CMGC | proposed | 99.0(threshold:4.7e-3) | 50.4 | 93.3 | 87.1 | 0.632 |
|  | GPS 3.0 | 99.0 | 16.3 | 89.2 | 68.4 | 0.298 |
|  | PPSP | 99.0 | 13.7 | 88.9 | 64.7 | 0.263 |
|  | NetPhos 3.1 | 99.0 | 15.0 | 89.1 | 66.7 | 0.281 |
|  | Wang et al. | 99.0 | 38.7 | 91.9 | 83.7 | 0.536 |

**Table S4**. Performance comparison with PTMPred for phosphorylation kinase groups by using all S and T site data.

| sites | Kinase groups | Methods | Sn(%) | Sp(%)* | Acc(%) | Pre(%) | MCC |
| --- | --- | --- | --- | --- | --- | --- | --- |
| T | AGC | proposed | 93.2 | 78.1 | 79.2 | 26.9 | 0.432 |
|  |  | PTMPred | 67.5 | 78.1 | 77.3 | 21.1 | 0.284 |
|  | CAMK | proposed | 40.8 | 97.1 | 94.9 | 34.8 | 0.351 |
|  |  | PTMPred | 26.3 | 97.1 | 94.5 | 26.3 | 0.235 |
|  | CK1 | proposed | 69.7 | 96.4 | 95.8 | 29.4 | 0.436 |
|  |  | PTMPred | 2.33 | 96.4 | 94.5 | 1.39 | -0.010 |
|  | CMGC | proposed | 86.7 | 92.1 | 91.5 | 59.6 | 0.674 |
|  |  | PTMPred | 73.7 | 92.1 | 89.9 | 55.6 | 0.584 |
|  | STE | proposed | 95.8 | 68.1 | 68.7 | 6.74 | 0.205 |
|  |  | PTMPred | 60.4 | 68.1 | 67.9 | 4.37 | 0.092 |
|  | TKL | proposed | 100 | 1.71 | 3.83 | 2.20 | 0.019 |
|  |  | PTMPred | 97.7 | 1.71 | 3.78 | 2.15 | -0.63 |
|  | Atypical | proposed | 100 | 3.38 | 6.09 | 2.89 | 0.031 |
|  |  | PTMPred | 98.2 | 3.38 | 6.04 | 2.84 | 0.015 |
|  | Other | proposed | 23.2 | 97.3 | 94.3 | 26.4 | 0.218 |
|  |  | PTMPred | 23.1 | 97.3 | 94.3 | 26.4 | 0.217 |
| S | AGC | proposed | 99.6 | 32.8 | 46.1 | 26.8 | 0.295 |
|  |  | PTMPred | 20.2 | 32.8 | 30.3 | 6.88 | -0.379 |
|  | CAMK | proposed | 98.8 | 14.4 | 20.8 | 8.77 | 0.103 |
|  |  | PTMPred | 100 | 14.4 | 21.0 | 8.87 | 0.113 |
|  | CK1 | proposed | 98.9 | 4.95 | 7.56 | 2.89 | 0.029 |
|  |  | PTMPred | 100 | 4.95 | 7.59 | 2.92 | 0.038 |
|  | CMGC | proposed | 100 | 11.6 | 29.1 | 21.9 | 0.159 |
|  |  | PTMPred | 96.1 | 11.6 | 28.3 | 21.2 | 0.102 |
|  | STE | proposed | 100 | 13.4 | 17.8 | 5.78 | 0.088 |
|  |  | PTMPred | 100 | 13.4 | 17.7 | 5.77 | 0.087 |
|  | TKL | proposed | 73.3 | 84.8 | 84.7 | 2.20 | 0.109 |
|  |  | PTMPred | 40.0 | 84.8 | 84.6 | 1.21 | 0.047 |
|  | Atypical | proposed | 73.1 | 95.1 | 93.6 | 51.2 | 0.579 |
|  |  | PTMPred | 37.2 | 95.1 | 91.2 | 34.6 | 0.312 |
|  | Other | proposed | 58.7 | 90.8 | 87.6 | 41.4 | 0.425 |
|  |  | PTMPred | 58.4 | 90.8 | 87.6 | 41.4 | 0.424 |

*: Unlike GPS and NetPhos, PTMPred does not give any prediction scores while provides binary prediction results, therefore we compared the Sn, Acc, Pre and MCC measurements at the same level of Sp value obtained by PTMPred. **Table S5**. Information of top ten ranked candidate sites for O-GalNAc.

| Ranking | UniProt ID | Protein name | position | Probability |
| --- | --- | --- | --- | --- |
| 1 | P38936 | CDKN1A | 98 | 0.776 |
| 2 | Q99700 | ATXN2 | 1150 | 0.748 |
| 3 | P35222 | CTNNB1 | 45 | 0.743 |
| 4 | Q9BRQ5 | ORAI3 | 191 | 0.731 |
| 5 | P55957 | BID | 64 | 0.723 |
| 6 | P24844 | MYL9 | 20 | 0.722 |
| 7 | Q8N3V7 | SYNPO | 120 | 0.703 |
| 8 | P13473 | LAMP2 | 207 | 0.702 |
| 9 | Q92934 | BAD | 99 | 0.701 |
| 10 | P52565 | ARHGDIA | 101 | 0.699 |

**Figure S1**. The ROC curves of different method for kinase group on S and T sites, respectively. Red, blue, purple and green lines represent the proposed method, GPS, PPSP and NetPhos, respectively. The upper part is the performance comparison on S sites and the lower part is the performance comparison on T sites. A, B and C are kinase groups AGC, CMGC and STE, respectively. D, E and F are kinase groups Atypical, CAMK and CK1, respectively.


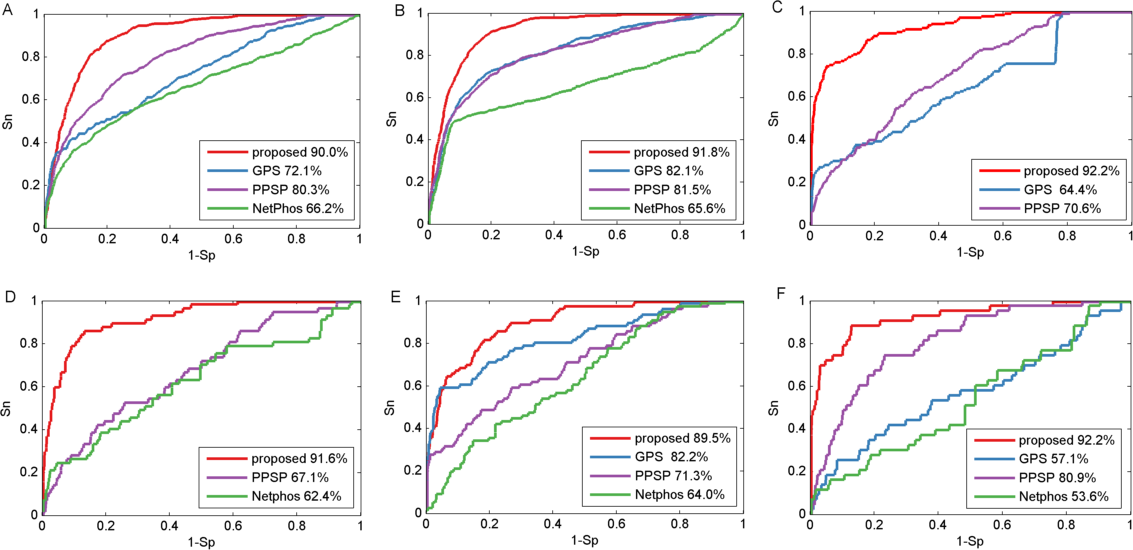


**Figure S2**. The ROC curves of different method for kinase group on Y sites. Red, blue, purple and green lines represent the proposed method, GPS, PPSP and NetPhos, respectively. A, B, C and D are kinase groups TK, CMGC, STE and Other, respectively.


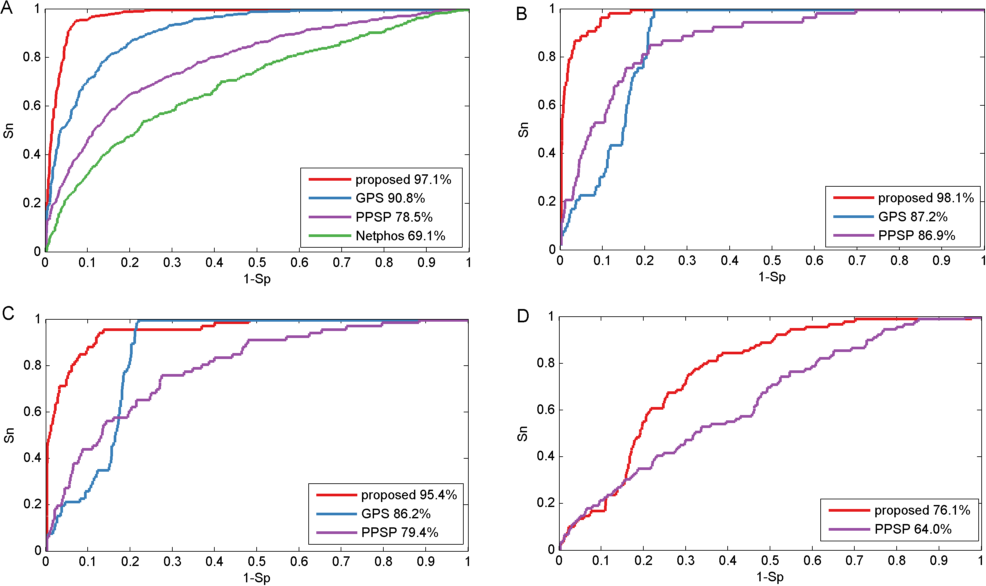


**Figure S3**. The fraction of predicted target sites for kinase group CAMK (A) and CMGC (B).


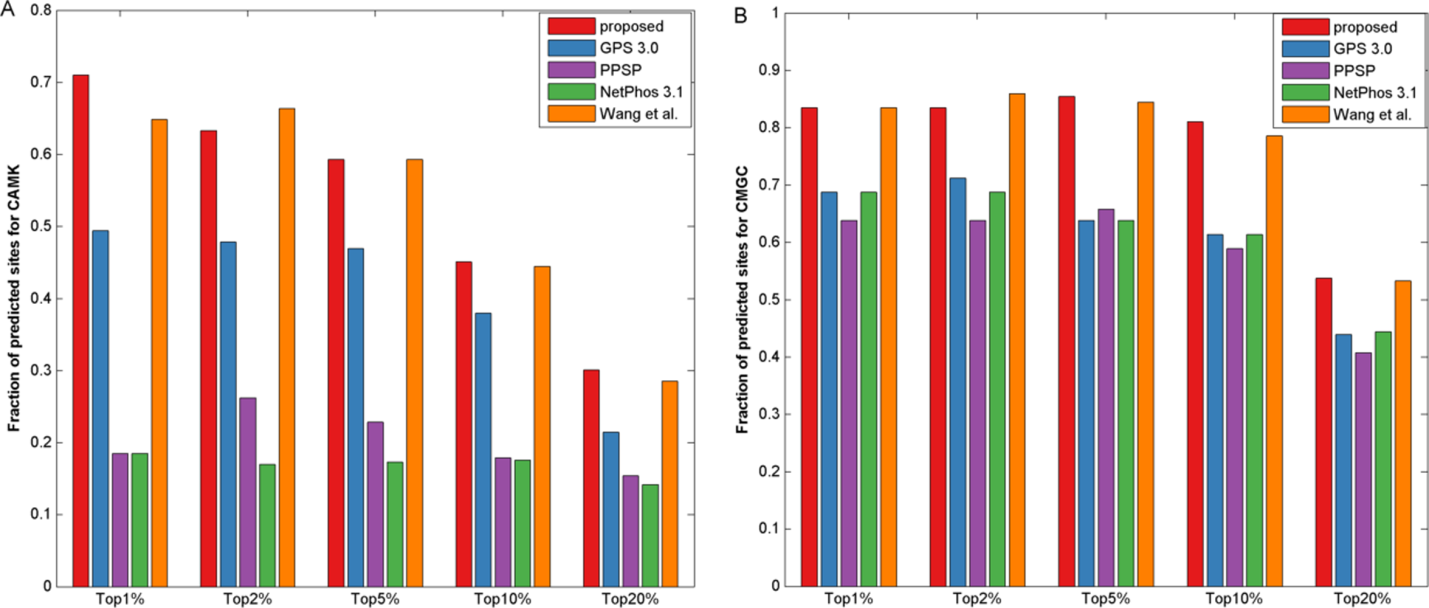

Supplement: Supplemental Information 1 [file peerj-05-3261-s001.docx]
